# Neuroprotective mechanism of salvianolic acid B against cerebral ischemia–reperfusion injury in mice through downregulation of TLR4, p‐p38MAPK, p‐JNK, NF‐κB, and IL‐1β

**DOI:** 10.1002/iid3.1030

**Published:** 2023-10-04

**Authors:** Xiu‐fen Zheng, Xiang‐jian Zhang, Li‐peng Dong, Jing‐ru Zhao, Cong Zhang, Rong Chen

**Affiliations:** ^1^ Department of Neurology Second Hospital of Hebei Medical University Shijiazhuang Hebei PR China; ^2^ Department of Pediatrics Tangshan Central Hospital Tangshan Hebei PR China; ^3^ Hebei Collaborative Innovation Center for Cardio‐cerebrovascular Disease Shijiazhuang Hebei PR China; ^4^ Hebei Key Laboratory of Vascular Homeostasis Shijiazhuang Hebei PR China; ^5^ Department of Neurology Hebei General Hospital Shijiazhuang Hebei PR China

**Keywords:** cerebral ischemia–reperfusion, inflammation, neuroprotection, salvianolic acid B

## Abstract

**Objective:**

Tissue injury and inflammation are two potential outcomes of cerebral ischemia–reperfusion (I/R) injury. Salvianolic acid B (Sal B), isolated from the roots of *Salvia miltiorrhiza*, is one of the major water‐soluble compounds with a wide range of pharmacological effects including antioxidant, anti‐inflammatory, antiproliferative, and neuroprotective effects. In the present study, we explored the neuroprotective effects and potential mechanisms of Sal B after I/R injury.

**Methods:**

We induced cerebral ischemia in male CD‐1 mice through transient (60 min) middle cerebral artery occlusion (tMCAO), and then injected Sal B (30 mg/kg) intraperitoneally. Neurological deficits, infarct volumes, and brain edema were assessed at 24 and 72 h after tMCAO. We detected the expression of Toll‐like receptor 4 (TLR4), phosphorylated‐p38 mitogen‐activated protein kinase (P‐p38 MAPK), phosphorylated c‐Jun amino (N)‐terminal kinases (p‐JNK), nuclear factor‐κB (NF‐κB), and interleukin‐1β (IL‐1β) in the brain tissue.

**Results:**

Compared with the tMCAO group, Sal B significantly improved neurological deficits, reduced infarct size, attenuated cerebral edema, and downregulated the expression of pro‐inflammatory mediators TLR4, p‐p38MAPK, p‐JNK, nuclear NF‐κB, and IL‐1β in brain tissue after I/R injury.

**Conclusion:**

We found that Sal B protects brain tissues from I/R injury by activating its anti‐inflammatory properties.

## 1 INTRODUCTION

Acute ischemic stroke is the leading cause of adult disability and the third leading cause of death in developed countries.^1^, ^2^ Ischemia–reperfusion (I/R) is a pathological state in which blood flow to an organ is temporarily cut off, followed by its rapid restoration and simultaneous reoxygenation. I/R can induce numerous secondary injuries, such as inflammation,^3^ which contributes to morbidity and mortality in ischemic cerebrovascular disease.^4^ The role of inflammation in the pathophysiology of reperfusion recovery after stroke is significant. Cerebral ischemia proteins like tumor necrosis factor alpha (TNF‐α) and interleukin 6 (IL‐6) can be elevated by I/R.^5^ Due to the established link between cerebral inflammation and I/R outcomes, research into anti‐inflammatory therapies to reduce I/R‐induced brain injuries has been continuously pursued.

Toll‐like receptors (TLRs) are innate immune receptors that recognize the pathogen‐associated molecular patterns in immune cells. They initiate a primary response to invading pathogens and mediate an adaptive immune response. A growing body of evidence suggests that TLRs are important mediators in cerebral ischemic injury.^4^ Several studies have demonstrated the importance of TLR‐4/nuclear factor‐κB (NF‐κB) signaling pathway in the pathogenesis of inflammation in cerebral I/R injury. Furthermore, I/R injury causes a substantial increase in the levels of TLR‐4 and other related protein factors.^6^ After I/R injury, TLRs activate downstream signaling pathways, including nuclear factor‐κB (NF‐κB), and mitogen‐activated protein kinase (MAPK) pathways, resulting in the production of pro‐inflammatory cytokines and chemokines, notably TNF and interleukin­‐1 (IL­1) which are important in inflammatory responses.^7^ Recent research has linked the pathophysiology of cerebral ischemia to the activation of TLRs, p38MAPK, NF‐κB, and c‐Jun amino (N)‐terminal kinases (JNK).^8^

Despite significant progress in understanding the pathophysiology of cerebral ischemia, there is currently no effective treatment for a stroke caused by acute ischemia. Salvianolic acid B (Sal B), isolated from the roots of *Salvia miltiorrhiza*, is a major water‐soluble compound widely used in traditional Chinese medicine. Studies have shown that Sal B has several pharmacological effects, including antioxidative,^9^ anti‐inflammatory, antiproliferative, and neuroprotective effects.^10^ However, the underlying mechanisms of its protective effects have not been clearly understood.

**Figure 1:**
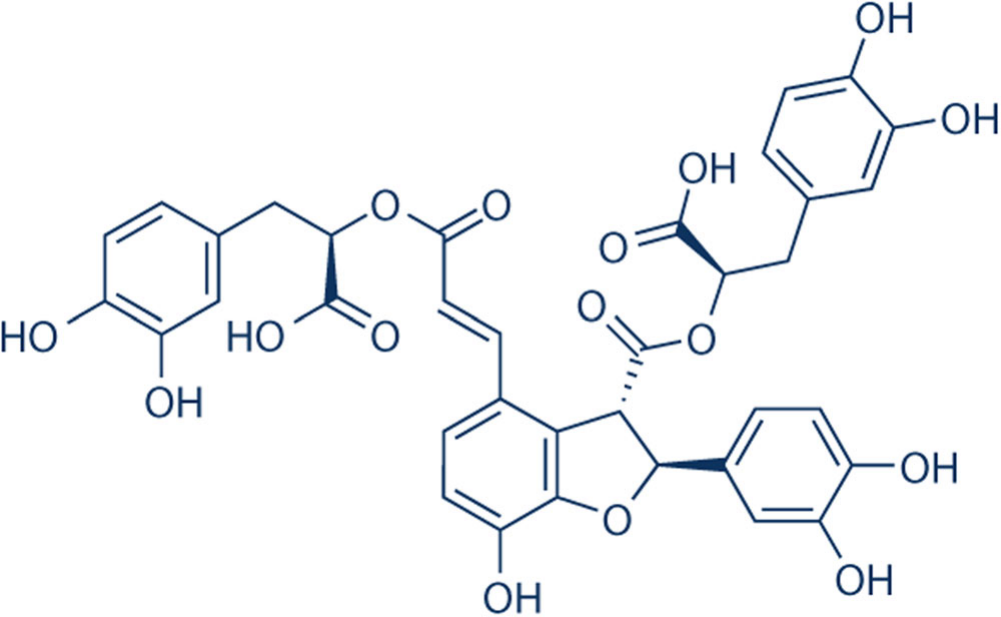
Chemical structure of Sal B.

In the present study, we used the mouse tMCAO model to illustrate the neuroprotective role of Sal B and its underlying mechanisms. We found that Sal B improved neurological deficits, reduced infarct volume, and decreased brain edema. Moreover, Sal B protected the brain against I/R injury via the TLR4/p38MAPK, JNK/NF‐κB/IL1‐β signal pathways. These findings point to the anti‐inflammatory properties of Sal B as a potential protective factor against cerebral ischemic stroke (Figure 1).

## 2 MATERIALS AND METHODS

### 2.1 Transient middle cerebral artery occlusion (tMCAO) model

Experimental procedures were performed in accordance with the National Institutes of Health Guide for the Care and Use of Laboratory Animals and were approved by the Institutional Care of Experimental Animals Committee (National Institutes of Health Guide for Care and Use of Laboratory Animals and European Communities Council Directive of 24 November 1986 (86/609/EEC)). Male CD1 mice (25–30 g VItal River Company) were acclimatized for a period of at least 3 days before the experiment. All mice had permissive access to food and water under controlled conditions (12/12 h light/dark with a humidity of 60 ± 5%, 22 ± 3°C). At first, animals were anesthetized with chloral hydrate (10%, 400 mg/kg, intraperitoneally). The mouse tMCAO model was established by right middle cerebral artery occlusion (60 min) followed by reperfusion, as described previously.^11^, ^12^ Body temperatures were monitored and maintained at 37 ± 0.5°C during surgery.

### 2.2 Experimental groups and drug administration

#### 2.2.1 Experimental groups

All the mice were randomly divided into three groups: (1) *Sham group*: animals underwent the same surgical procedures without inserting a filament and received an equal volume of 0.9% NaCl; (2) *tMCAO group*: animals received tMCAO and equal volume of 0.9% NaCl; (3) *Sal B group*: animals underwent tMCAO and received Sal B (purity 98%, Hengyuanqitian).

#### 2.2.2 Drug administration

Sal B was diluted in saline to a concentration of 0.3%, and mice in the Sal B group received an intraperitoneal injection of 30 mg/kg Sal B immediately following MCAO.^13^ Mice in the tMCAO and Sham groups received equivalent volumes of 0.9% saline in the same manner.

### 2.3 Evaluation of neurological deficit

Neurological deficit scores (NDS) were assessed at 24 and 72 h after tMCAO by a researcher who was blinded to the experimental group (*n* = 10 per group per time point). The assessment was as per a modified scoring system based on that developed by Longa et al. (1989): 0—no deficit; 1—difficulty in fully extending the contralateral forelimb; 2—inability to extend the contralateral forelimb; 3—mild circling to the contralateral side; 4—severe circling; and 5—no spontaneous activity or falling to the contralateral side.^14^

### 2.4 Measurement of brain water content

We measured brain water content using the standard wet–dry method at 24 and 72 h after ischemia.^8^, ^15^ The brains were swiftly deleted after the mice (*n* = 6 per group per time point) were anesthetized with 10% chloral hydrate and euthanized. The frontal lobe was sectioned off into 3‐mm‐thick coronal slices, and the halves were labeled as ipsilateral and contralateral hemispheres. The wet weight of the samples was immediately weighed on an electronic balance and then dried in an oven at 100°C for 24 h to obtain the dry weight. The formula for calculating the water content of the brain was (wet weight − dry weight)/wet weight ×  100%.

### 2.5 Measurement of brain infarct volume

We determined infarct volume using 2,3,5‐triphenyltetrazolium chloride (TTC) staining at 24 and 72 h after ischemia (*n* = 10 per group per time point). The brain tissue was sectioned off into five slices of 2 mm thickness and stained with a 2% solution of TTC at 37°C for 20 min,^16^ followed by fixation with 4% paraformaldehyde. We photographed the brain sections stained by TTC and analyzed the digital images using an image analysis software (Image‐Pro Plus 5.1; Media Cybernetics, Inc.).

We calculated the lesion volume by multiplying the area by the thickness of the slices. To compensate for the effect of brain edema, we calculated the percentage hemisphere lesion volume (%HLV) using the following formula^17^: (%HLV)  = {[total infarct volume − (volume of intact ipsilateral hemisphere − volume of intact contralateral hemisphere)]/contralateral hemisphere volume} × 100%.

### 2.6 Immunohistochemical staining

We investigated the impact of Sal B on the positive cell expression of TLR4, p‐p38MAPK, p‐JNK, NF‐κB, and IL1‐β in ischemic hemispheres using immunohistochemistry at 24 and 72 h after tMCAO.^18^ Briefly, brains were fixed in 4% paraformaldehyde in phosphate‐buffered saline (PBS; 0.01 M, pH 7.4) over 24 h at 4°C and then dehydrated in a graded series of alcohols and embedded in paraffin. Brain section (5‐μm‐thick) was blocked by 3% H_2_O_2_ and 3% normal goat serum to eliminate endogenous peroxidase activity, then incubated with TLR4 rabbit polyclonal antibody (1:50, Bioworld Biotechnology) and NF‐κB P65 rabbit polyclonal antibody (1:100, Bioworld Biotechnology), p‐p38MAPK rabbit polyclonal antibody (1:100, Cell Signaling Technology), p‐JNK rabbit polyclonal antibody (1:50, Cell Signaling Technology), and IL1‐β rabbit polyclonal antibody (1:100, Abcam Biotechnology) overnight in 0.01 mol/L PBS at 4°C.

We used the secondary antibodies, secondary biotinylated conjugates, and diaminobenzidine from the kit (Zhongshan Biology Technology Company) to visualize the signals. Five visual fields of the ischemic region of the infarct were selected, and the immunoreactive cells were counted under a ×400 light microscope (*n* = 3 per group per time point). For statistical analysis, the number of immunopositive cells in a mouse was represented by its mean.

### 2.7 Western blot analysis

We extracted proteins from the cortex at 24 and 72 h after cerebral ischemia (*n* = 6 per group per time point) using the Total Protein Extraction Kit and the Nucleocytolysis Extraction Kit (Applygen Technologies Inc.) according to the manufacturer's protocol.^19^ Protein concentrations were determined using the BCA Protein Assay Kit (Novagen). An equivalent amount of 20 μg total protein samples as well as 30 μg nuclear protein samples were separated using SDS/PAGE and transferred onto PVDF membranes. After 30 min at room temperature, we diluted anti TLR4 antibody (1:200) and anti‐NF‐κB P65 antibody (1:500), anti‐p38MAPK, anti‐p‐p38MAPK, anti‐JNK, anti‐p‐JNK antibodies (1:500), and anti‐IL1‐β antibody (1:1000) with 3% BSA‐TBST for 10 min, and then incubated it overnight at 4°C. GAPDH (1:1000; Tiandeyue Biotechnology) was used for an internal control. After being washed with TBST (5 min × 3), all membranes were incubated in 5% nonfat dry‐TBST containing HRP labeling second antibodies (goat anti‐rabbit IgG; Tiandeyue Biotechnology) for 40 min at room temperature, and then washed with TBST (3 min × 6). We used ECL developer, and analyzed the relative density of each band using TotalLab Quant.

### 2.8 Polymerase chain reaction (qRT‐PCR)

qRT‐PCR was used to analyze the mRNA expression levels of TLR4, NF‐κB, and IL‐1β at 24 and 72 h after tMCAO (*n* = 6 per group per time point). Total RNA from the ischemic region cortex was isolated using Trizol reagent (Tiangen Biotechnology) and reverse‐transcribed into cDNA using PrimeScript™ RT reagent Kit with gDNA Eraser (TaKaRa). We carried out amplification and detection with the ABI 7500 Real‐Time PCR system (Applied Biosystems) using SYBR® Premix Ex Taq™ II (Tli RNaseH Plus), ROX plus (TaKaRa). Results for each sample were collected at least three times. Relative abundance of mRNA from the prospective target genes was calculated via $2^{-\Delta \Delta C_{T}}$ method after normalization to ACTB RNA.

The primers were as follows: TLR4 forward, 5′‐GAG GAC TGG GTG AGA AAT GAG‐3′, reverse, 5′‐GTT GGC AGC AAT GGC TAC AC‐3′; NF‐κB forward, 5′‐AAT GGC TAC ACA GGA CCA GGA ACA‐3′, reverse, 5′‐GGA TTC GCT GGC TAA TGG CTT GCT C‐3′; IL‐1β forward, 5′‐TGC CAC CTT TTG ACA GTG ATG A‐3′, reverse, 5′‐TGT GCT GCT GCG AGA TTT GA‐3′; the housekeep gene ACTB forward, 5′‐GCC TTC CTT CTT GGG TAT‐3′, reverse, 5′‐GGC ATA GAG GTC TTT ACG G‐3′.

### 2.9 Statistical analysis

We analyzed all data using SPSS13.0 software. Quantitative data are expressed as mean ± SD. We used one‐way analysis of variance (ANOVA) for statistical comparisons and the Student–Newman–Keuls method for multiple comparisons. We used the independent‐samples *t*‐test to analyze infarct volume. We used the Mann–Whitney *U* test for comparisons of neurologic deficits between two groups. The difference was considered statistically significant if *p* < .05.

## 3 RESULTS

### 3.1 Sal B ameliorated neurologic deficits after tMCAO

**Figure 2:**
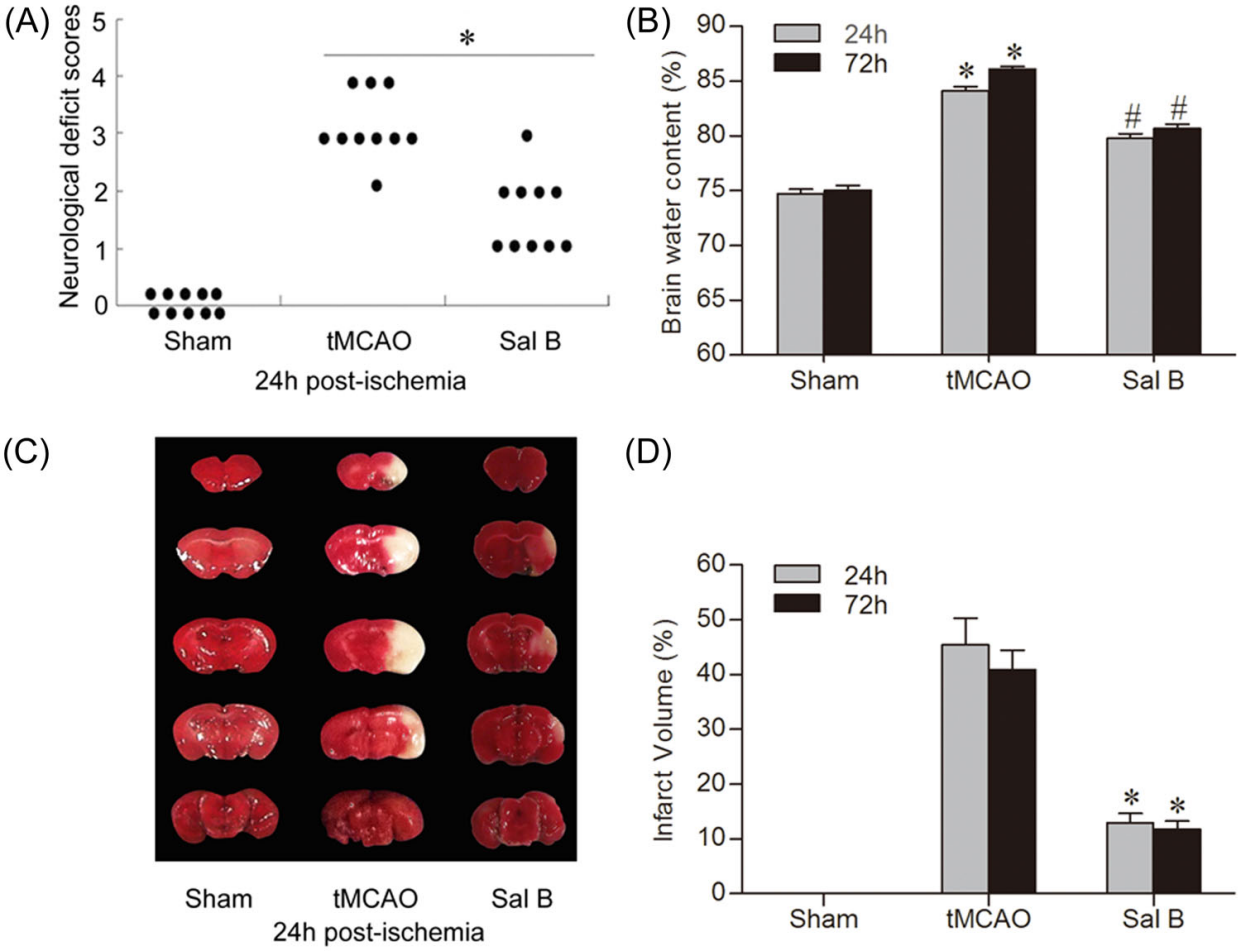
Effect of Sal B on mice brains after transient middle cerebral artery occlusion (tMCAO). Graphs show the neurological deficit scores at 24 h after tMCAO. Each circle represents the score for a mouse. Compared with the tMCAO group, the neurological deficit scores are significantly lower in the Sal B group (30 mg/kg) at 24 h (A); *p < 0.05. Sal B significantly reduced brain edema and infarct size at 24 and 72 h after tMCAO (B, D); *p < .05 versus Sham group; ^#^ *Ρ* < 0.05 versus tMCAO group. Representative images of TTC staining at 24 h after ischemia stroke (C).

We evaluated the extent of the neurological deficit and scored it on a 6‐point scale at 24 and 72 h after tMCAO. We used the Mann–Whitney *U* test for statistical analysis (Figure 2A). We found that the mice in the Sham group did not show any neurological deficit. Compared with the tMCAO group, mice in the Sal B group showed a significant improvement in neurologic function scores at 24 and 72 h after ischemia (*p* < .05 for all).

### 3.2 Sal B reduced brain edema

We measured cerebral water content using the wet–dry method. Cerebral edema in the ischemic hemisphere of each group at 24 and 72 h is shown in Figure 2B. Brain water content in the ischemic hemisphere was significantly increased at all time points in the tMCAO group compared with the Sham group (24 h: 74.74% ± 0.96% vs. 84.11% ±  0.99%; 72 h: 75.05% ± 1.01% vs. 86.12% ± 0.53%, respectively; *p* < .05 for all). Compared with the tMCAO group, Sal B significantly reduced the brain water content in ischemic hemispheres (24 h: 79.99% ± 0.98% vs. 84.11% ± 0.99%; 72 h: 80.66% ± 1.02% vs. 86.12% ± 0.53%, respectively; *p* < .05 for all).

### 3.3 Sal B reduced infarct volume

We detected brain infarct volume using TTC dyeing at 24 and 72 h after ischemia (Figure 2C,D). After TTC staining, the normal tissue was dark red while the infarcted area was light gray. No infarcts occurred in the Sham group; however, extensive lesions were found in the cerebral cortex and striatum in the tMCAO group. Compared with the tMCAO group, infarct size in the Sal B group was significantly reduced at 24 and 72 h after ischemia (24 h: 45.39% ± 11.98% vs. 12.92% ± 4.17%; 72 h: 40.92% ± 8.63% vs. 11.76% ± 3.60%, respectively; *p* < .05 for all).

### 3.4 Sal B inhibited the positive cells expression of TLR4, p‐p38MAPK, p‐JNK, NF‐κB, and IL1‐β after tMCAO

**Figure 3:**
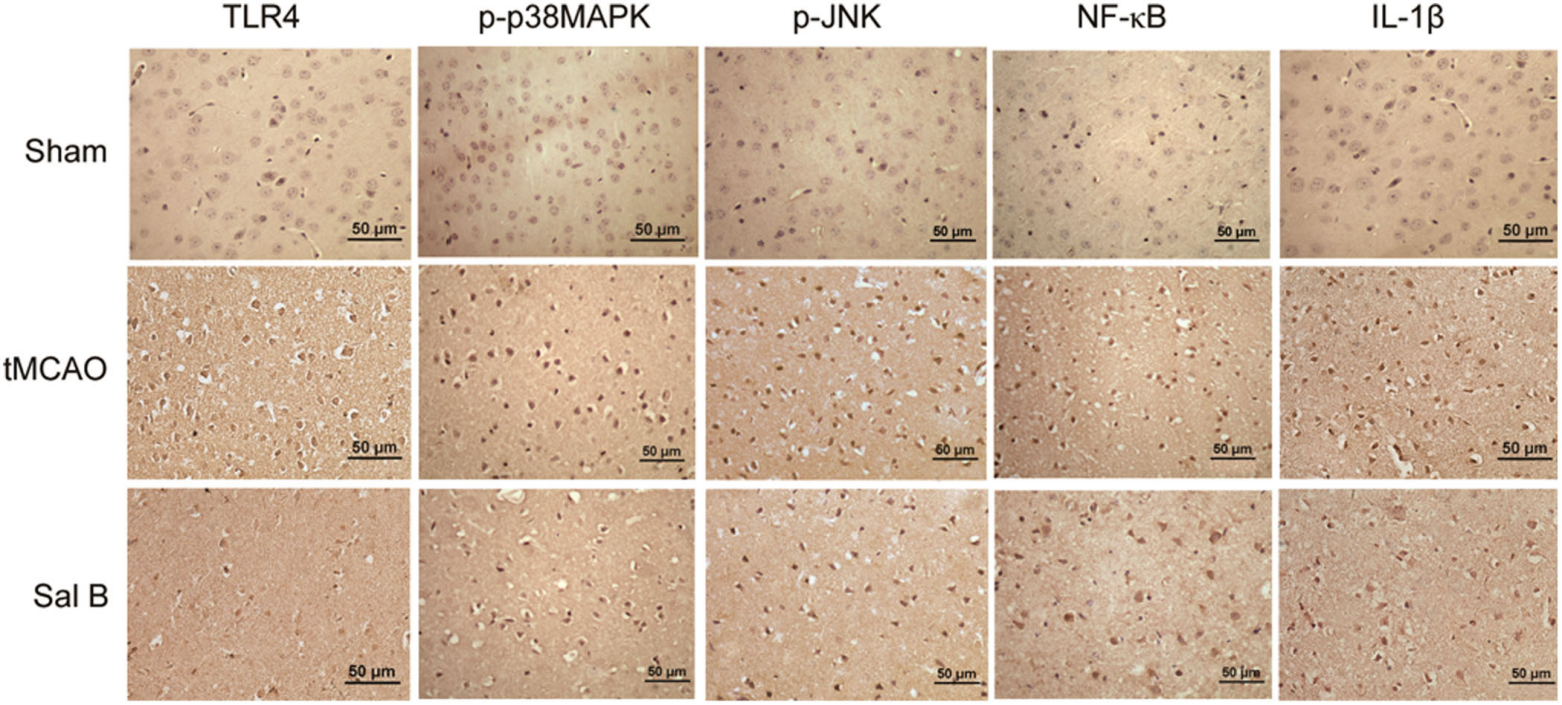
Representative images for immunohistochemical staining of TLR4, p‐p38MAPK, p‐JNK, NF‐κB, and IL1‐β at 24 h after transient middle cerebral artery occlusion (tMCAO) in different groups; scale bar, 50 µm; magnifications of the microphotograph, ×400.

**Figure 4:**
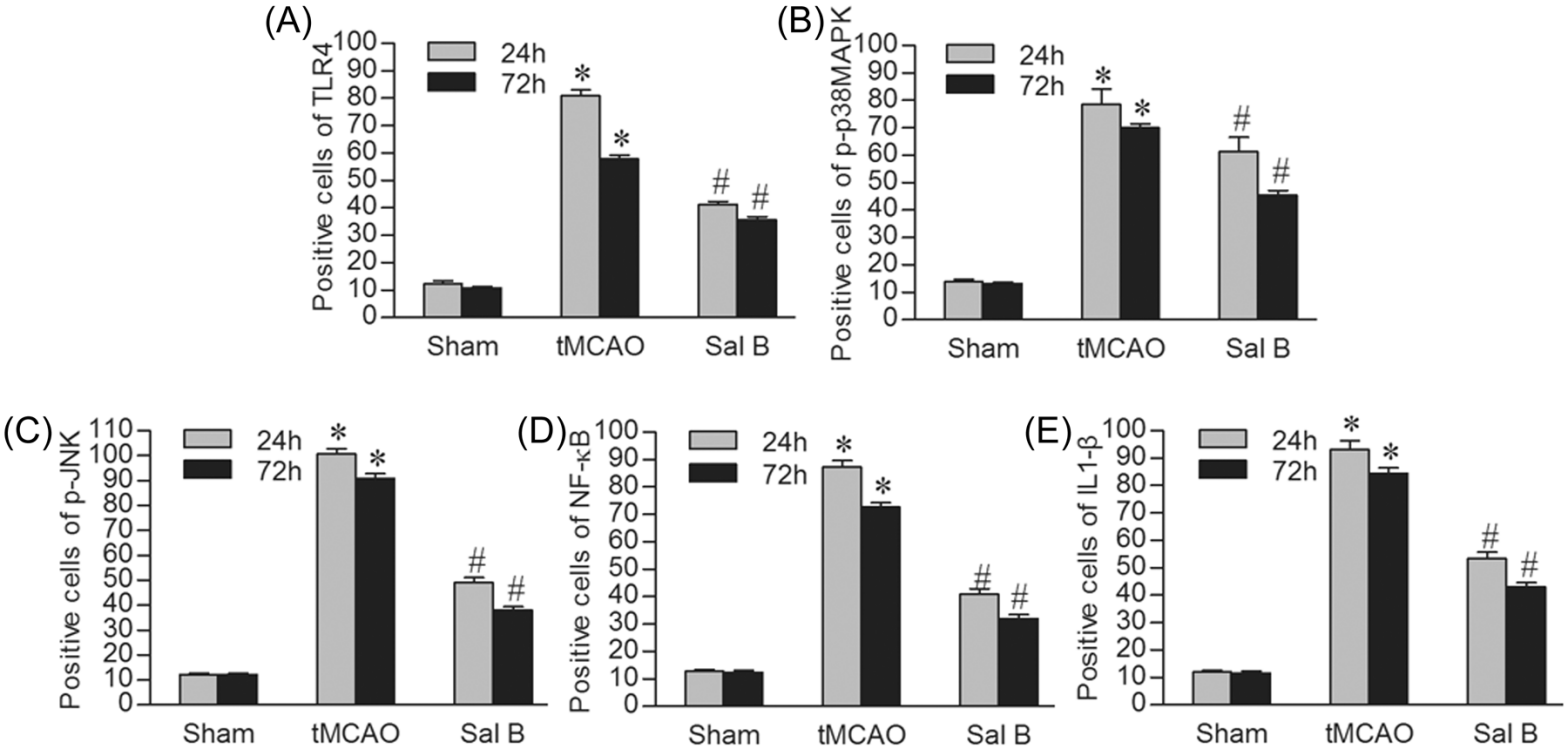
Effect of Sal B on the number of positive cells stained by TLR4, p‐p38MAPK, p‐JNK, NF‐κB, and IL1‐β after transient middle cerebral artery occlusion (tMCAO). As shown in the bar graphs A–E, compared with the Sham group, the number of positive cells stained by TLR4 (A), p‐p38MAPK (B), p‐JNK (C), NF‐κB (D), and IL1‐β (E) is higher in the tMCAO group at 24 and 72 h, while the positive cells stained by examined proteins in the Sal B group are significantly lower compared with the tMCAO group at any time point. *p < .05 versus Sham group; ^#^ *p* < 0.05 versus tMCAO group.

We investigated the effect of Sal B on the expression of TLR4, p‐p38MAPK, p‐JNK, NF‐κB, and IL1β‐positive cells in ischemic hemispheres using immunohistochemistry at 24 and 72 h after tMCAO. The results of immunohistochemical staining of TLR4, p‐p38MAPK, p‐JNK, NF‐κB, and IL1‐β in each experimental group at 24 h after ischemia are shown in Figure 3. We manually counted the positively stained cells and performed intergroup comparisons. We found that there were few positive cells stained by TLR4, p‐p38MAPK, p‐JNK, NF‐κB, and IL1‐β in the cortex in the Sham group. The tMCAO group had a higher number of positive cells stained by these proteins at 24 and 72 h compared to the Sham group (*p* < .05), while the expression levels of TLR4, p‐p38MAPK, p‐JNK, NF‐κB, and IL1‐β in the Sal B group were significantly lower compared with the tMCAO group at any time point (*p* < .05) (Figure 4A–E).

### 3.5 Sal B inhibited the protein expression of TLR4, p‐p38MAPK, p‐JNK, NF‐κB, and IL1‐β

**Figure 5:**
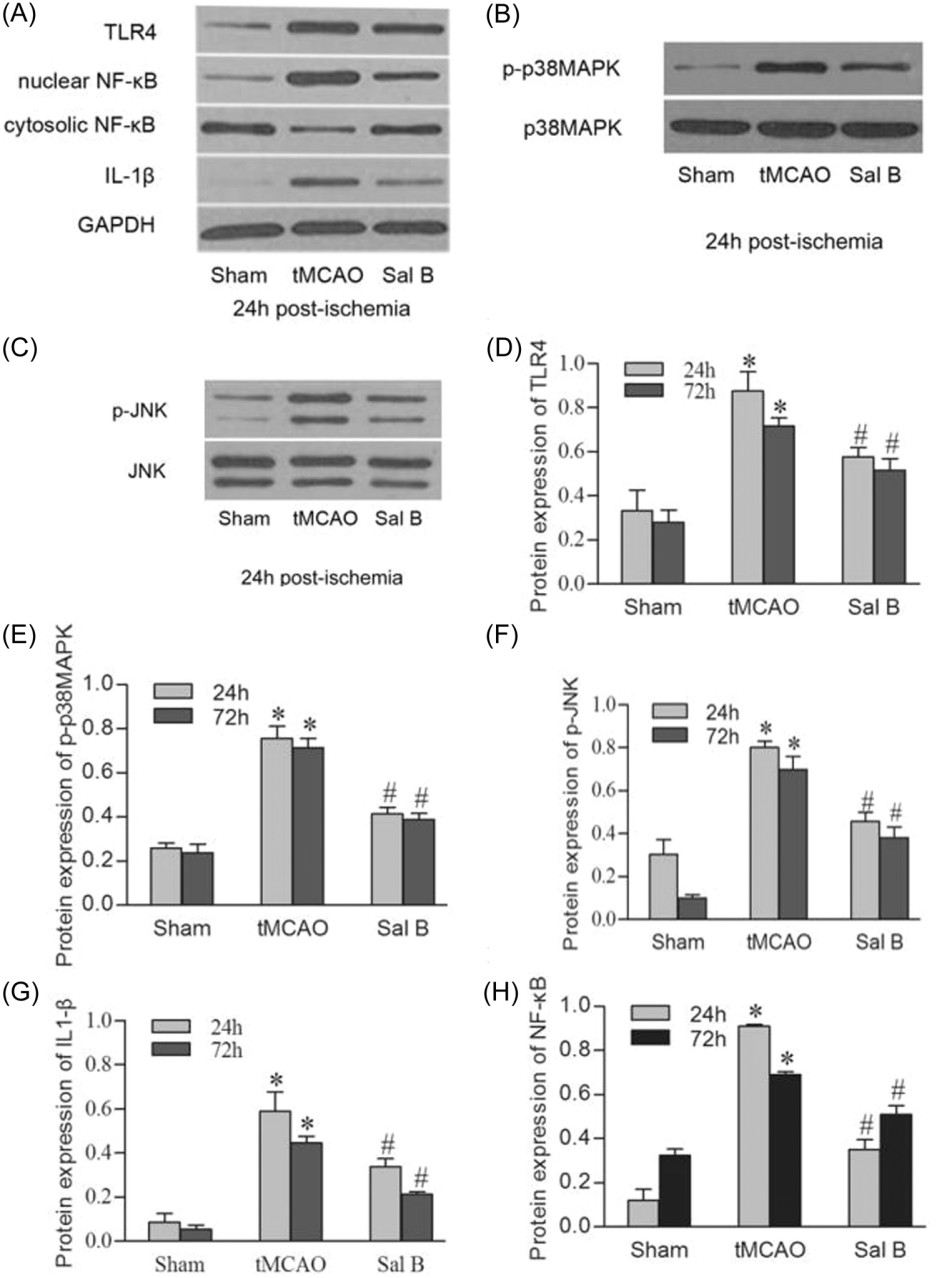
Effect of Sal B on protein expression of TLR4, p‐p38MAPK, p‐JNK, NF‐κB, and IL‐1β after transient middle cerebral artery occlusion (tMCAO). Representative photographs of western blot analysis show the influence of Sal B on the expression TLR4, NF‐κB, IL1‐β (A), total p38MAPK, and p38MAPK (B), p‐JNK, and total JNK (C) in the cerebral cortex at 24 h after tMCAO. As shown in the bar graphs, western blot analysis also shows a significant increase of the protein expression of TLR4 (D), p‐p38MAPK (E), p‐JNK (F), IL‐1β (G), and nuclear NF‐κB (H) in the tMCAO group at 24 and 72 h compared with the Sham group. Compared with the tMCAO group, Sal B significantly attenuated the protein expression of TLR4, p‐p38MAPK, p‐JNK, nuclear NF‐κB, and IL‐1β at 24 and 72 h after tMCAO. *p < .05 versus sham group; ^#^ *p* < 0.05 versus tMCAO group.

We used western blot analysis to analyze TLR4, p‐p38MAPK, p‐JNK, nuclear NF‐κB, and IL1‐β protein expression in the ischemic hemispheres at 24 and 72 h after ischemia. Representative photographs of TLR4, total p38MAPK/p‐p38MAPK, total JNK/p‐JNK, nuclear NF‐κB/cytosolic NF‐κB, and IL1‐β in each experimental group at 24 h after ischemia are shown in Figure 5A–C. Similar to the immunohistochemistry results, western blot analysis showed a significant increase of TLR4, p‐p38MAPK, p‐JNK, and IL‐1β in the tMCAO group at 24 and 72 h compared with the Sham group (*p* < .05 for all). Sal B significantly attenuated the protein expression of TLR4, p‐p38MAPK, p‐JNK, and IL‐1β compared with the tMCAO group at 24 and 72 h after cerebral ischemia (*p* < .05 for all). We also found a significant increase in nuclear protein NF‐κB, but a significant decrease in cytosol at 24 and 72 h after cerebral ischemia. Sal B inhibited the nuclei translocation of NF‐κB and resulted in a significant decrease in nuclear protein NF‐κB (*p* < .05 for all) (Figure 5D–H).

### 3.6 Sal B inhibited the mRNA expression of TLR4, NF‐κB, and IL1‐β

**Figure 6:**
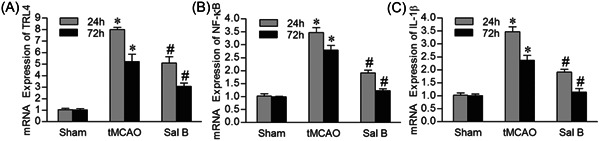
Effect of Sal B on mRNA expression of TLR4, NF‐κB, and IL‐1β after transient middle cerebral artery occlusion (tMCAO). As shown in the bar graphs (A–C), mRNA level of TLR4 (A), NF‐κB (B), and IL‐1β (C) are increased in the tMCAO group after ischemia at 24 and 72 h, while Sal B significantly reduced mRNA expression level of these proteins. *p < .05 versus sham group; ^#^ *p* < 0.05 versus tMCAO group.

We further investigated the mRNA expression of TLR4, NF‐κB, and IL‐1β in each group using qRT‐PCR. Consistent with immunohistochemistry and western blot analysis, compared with the Sham group, the mRNA levels of TLR4, NF‐κB, and IL‐1β were significantly increased in the tMCAO group at 24 and 72 h (*p* < .05). Compared with the tMCAO group, the expression of TLR4, NF‐κB, and IL‐1β was decreased in the presence of Sal B at both 24 and 72 h (*p* < .05) (Figure 6A–C).

## 4 DISCUSSION

Arterial blood occlusion can lead to a serious imbalance between metabolic supply and demand. Sequential reperfusion injury has been consistently associated with a deterioration of tissue injury and a profound inflammatory response.^20^ Hence, in the early stage of ischemia, anti‐inflammatory treatments can alleviate I/R‐induced inflammation and improve the outcome in stroke patients.^21^ It has been demonstrated that the TLR‐MyD88 association activates MAPKs and NF‐κB pathway cascades, which are necessary for the expression of inflammatory mediators such as IL‐1β.^22^ In this study, we investigated the neuroprotective role of Sal B against ischemic brain damage using a mouse tMCAO model as a classical model that mimics human stroke.^11^ Sal B improved neurological dysfunction, reduced the size of cerebral infarcts and cerebral edema, and inhibited the inflammatory response following cerebral I/R injury by downregulating TLR4, p‐p38MAPK, p‐JNK, nuclear NF‐κB, and IL‐1β production.

TLRs are coreceptors of the innate immune response and are triggered when infectious microbes invade mammals. They are also activated by host‐derived molecules. Studies have demonstrated that TLR4 plays an important role in the innate immunity response of the central nervous system,^1^ and participates in cerebral damage caused by ischemic strokes.^23^ Multiple studies have shown that TLR4‐deficient or TLR4‐mutant mice exhibit a protective effect against ischemic brain damage by decreasing the infarct size and pro‐inflammatory cytokine secretion.

MAPKs, the downstream signaling pathways of TLRs, are evolutionarily conserved enzymes. Once activated, MAPKs phosphorylate specific serine and threonine residues of target substrates, which include many transcription factors and other protein kinases.^24^ JNK and p38MAPK, two major MAPK pathways, which are also called stress‐activated protein kinase pathways, are activated by environmental and genotoxic stresses, and play key roles in inflammation.^25^ They are activated by phosphorylation and cause the transcription of several cytokines to produce inflammatory mediators such as tumor necrosis fctor‐α (TNF‐α) and interleukins (ILs).^26^, ^27^, ^28^

Previous studies have shown that cerebral ischemia is closely related to p38MAPK and JNKs.^8^, ^29^ As p38MAPK activation is involved in ischemia‐induced neurological injury, blocking it may protect brain tissue from ischemia damage by decreasing the production of inflammatory mediators and blocking the inflammatory process.^8^ Additionally, inhibiting JNK activation protects against cell death following ischemia. The importance of JNKs and p38MAPK in inflammation makes them promising therapeutic targets.^30^

As nuclear targets of MAPKs pathways, NF‐κB occupies a key position in the inflammatory response process. When cells are activated by signals, they respond by activating NF‐κB by promoting the phosphorylation and degradation of I‐κB.^31^ Then, the activated NF‐κB is translocated into the nucleus, resulting in the transcriptional expression of genes correlated with cellular growth properties.^32^ Liu et al.^33^ reported that elevated NF‐κB contributes to neurological injury induced by ischemia. Synthetic NF‐κB inhibitors were found to decrease infarct size in the treatment of permanent ischemia.^23^

Most inflammatory responses are mediated by cytokines, which may lead to ischemic brain damage. Members of the IL‐1 family are expressed poorly in healthy brain tissue but are dramatically upregulated during ischemia. Intraventricular injection of recombinant IL‐1β after MCAO was found to aggravate brain edema and increase the infarct volume and the influx of neutrophils,^34^ while IL‐1‐deficient mice showed smaller infarct volumes compared with wild‐type mice.^35^

The results of our study revealed that after cerebral ischemia, there is a significant increase in NDS, cerebral edema, and infarct volume, and TLR4, p‐p38MAPK, p‐JNK, and IL‐1β expression were all upregulated in the initial phase after tMCAO. Furthermore, NF‐κB expression increased in the nucleus and decreased in the cytoplasm after tMCAO. This finding lends credence to the idea that these cytokines play a significant role in the early post‐transient ischemia period, and intervening in their regulation can bring great benefits in brain I/R injury.

Sal B, a hydrophilic caffeic acid derivative from *S. miltiorrhiza*, represents the most abundant and bioactive component of *S. miltiorrhiza*.^36^ Multiple biological effects of Sal B have been investigated. It has been reported that Sal B has an anti‐fibrotic effect in TGF‐β1‐stimulated human hepatic stellate cell line by inhibition of ERK and p38 MAPK signaling.^35^ Sal B also protects neonatal cardiomyocyte injury induced by LPS by inhibiting the TLR4–NF‐κB–TNFα pathway.^36^ Lee et al.^10^ confirmed that Sal B facilitates neuroprotection via anti‐inflammatory and antioxidative effects by ameliorating memory impairment, reducing the number of activated microglia and astrocytes, and inducing nitric oxide synthase and cyclooxygenase‐2 expression levels in a mouse model of Alzheimer's disease. The expression of pro‐inflammatory factors such as IL‐1β, TNF‐α, and NF‐κB was significantly increased after traumatic brain injury or spinal cord injury in animal models, and treatment with Sal B greatly attenuated this upregulation.^37^

In light of these results, we speculate that TLR4/p38MAPK, JNK/NF‐κB/IL1‐β signaling pathway may be involved in the therapeutic effect of Sal B in our mouse model of tMCAO. Our results show that Sal B has a significant neuroprotective effect against brain I/R injury by lowering cerebral infarction, neurological scores, and cerebral edema following tMCAO. In addition, we investigated the anti‐inflammatory properties of Sal B. Following cerebral ischemia, we found that Sal B significantly reduced TLR4, p‐p38MAPK, p‐JNK, IL‐1β expression, and nuclear translocation of NF‐κB. These findings demonstrate the neuroprotective potential of Sal B due to its anti‐inflammatory properties. However, we acknowledge the need for additional research into the effect of Sal B on cerebral I/R in larger animal models and in other higher‐order species. More study is required to determine the full scope of the mechanism of action of Sal B in terms of anti‐inflammatory and neuroprotective effects.

## 5 CONCLUSION

Our results provide evidence that Sal B mitigates the effects of cerebral I/R injury. This effect may be due to the downregulation of the expression of TLR4, NF‐κB, p‐p38MAPK, p‐JNK, and IL‐1β. Sal B shows great potential as a future protective agent against cerebrovascular disease.

## AUTHOR CONTRIBUTIONS

*Conception and design of the research*: Xiu‐fen Zheng and Xiang‐jian Zhang. *Acquisition of data*: Xiu‐fen Zheng, Li‐peng Dong, and Jing‐ru Zhao. *Analysis and interpretation of the data*: Li‐peng Dong, Jing‐ru Zhao, Cong Zhang, and Rong Chen. *Statistical analysis*: Xiu‐fen Zheng, Rong Chen, and Cong Zhang. *Obtaining financing*: Xiang‐jian Zhang. *Writing of the manuscript*: Xiu‐fen Zheng. *Critical revision of the manuscript for intellectual content*: Xiang‐jian Zhang and Rong Chen. All authors read and approved the final draft.
