# EXPRESSION OF CONCERN: Neuroprotective Mechanism of Salvianolic Acid B Against Cerebral Ischemia–Reperfusion Injury in Mice Through Downregulation of TLR4, p‐p38MAPK, p‐JNK, NF‐**κB**, and IL‐1β

**DOI:** 10.1002/iid3.70314

**Published:** 2025-12-30

**EXPRESSION OF CONCERN**: X. Zheng, X. Zhang, L. Dong, J. Zhao, C. Zhang and R. Chen, “Neuroprotective Mechanism of Salvianolic Acid B Against Cerebral Ischemia–Reperfusion Injury in Mice Through Downregulation of TLR4, p‐p38MAPK, p‐JNK, NF‐κB, and IL‐1β,” *Immunity, Inflammation and Disease* 11, no. 10 (2023): e1030, https://doi.org/10.1002/iid3.1030.

This Expression of Concern is for the above article, published online on 04 October 2023 in Wiley Online Library (wileyonlinelibrary.com), and has been issued by agreement between the journal Editor‐in‐Chief, Marc Veldhoen; and John Wiley & Sons Ltd. The Expression of Concern has been agreed upon due to inconsistencies and discrepancies identified in Figure 2. Although the authors provided some raw data corresponding to the study, the data supplied was incomplete. Additionally, the authors were unable to provide documentation of ethics approval granted prior to the commencement of the study, as required. Instead, retrospective approval was obtained. The journal is issuing this expression of concern to alert readers.

